# A Cucumber *DELLA* Homolog *CsGAIP* May Inhibit Staminate Development through Transcriptional Repression of B Class Floral Homeotic Genes

**DOI:** 10.1371/journal.pone.0091804

**Published:** 2014-03-14

**Authors:** Yan Zhang, Bin Liu, Sen Yang, Jingbo An, Chunhua Chen, Xiaolan Zhang, Huazhong Ren

**Affiliations:** Department of Vegetable Science, College of Agronomy and Bio-technology, China Agricultural University, Beijing, P.R. China; Department of Vegetable Science, Beijing Key Laboratory of Growth and Developmental Regulation for Protected Vegetable Crops, China Agricultural University, Beijing, P.R. China; Institute of Genetics and Developmental Biology, Chinese Academy of Sciences, China

## Abstract

In hermaphroditic *Arabidopsis*, the phytohormone gibberellin (GA) stimulates stamen development by opposing the *DELLA* repression of B and C classes of floral homeotic genes. GA can promote male flower formation in cucumber (*Cucumis sativus* L.), a typical monoecious vegetable with unisexual flowers, and the molecular mechanism remains unknown. Here we characterized a *DELLA* homolog *CsGAIP* in cucumber, and we found that *CsGAIP* is highly expressed in stem and male flower buds. *In situ* hybridization showed that *CsGAIP* is greatly enriched in the stamen primordia, especially during the hermaphrodite stage of flower development. Further, *CsGAIP* protein is located in nucleus. *CsGAIP* can partially rescue the plant height, stamen development and fertility phenotypes of *Arabidopsis rga-24/gai-t6* mutant, and ectopic expression of *CsGAIP* in wide-type *Arabidopsis* results in reduced number of stamens and decreased transcription of B class floral homeotic genes *APETALA3* (*AP3*) and *PISTILLATA* (*PI*). Our data suggest that monoecious *CsGAIP* may inhibit staminate development through transcriptional repression of B class floral homeotic genes in *Arabidopsis*.

## Introduction

Gibberellins (GAs) are one class of tetracyclic diterpenoid phytohormones that play essential roles in diverse aspects of plant growth and development, including seed germination, hypocotyl elongation, root growth, stem elongation, leaf expansion, trichome formation, floral induction, flower development, and fruit development [1], in which, floral induction and flower development are the most important events regulated by GA [2]. GA content has been shown to increase dramatically before anthesis in flowers of both monocotyledonous and dicotyledonous species, such as barely (*Hordeum vulgare*), rice (*Oryza sativa*), *Mirabilis jalapa* and *Pharbitis*, implying that GA may be required for flower opening [3]. GA treatment, however, has distinct, even opposite effects on flower development in different species. For example, GA application can promote staminate development in *Arabidopsis*, rice and tomato (*Solanum lycopersicum*), whereas stimulate pistillate development in castor bean (*Ricinus communis*), *Hyoscyamus* and maize (*Zea mays*) [3].

Several key enzymes have been identified to be involved in GA biosynthesis, such as copalyl diphosphate synthase (*CPS*), ent-kaurene synthase (*KS*), ent-kaurene oxidase (*KO*) and ent-kaurenoic acid oxidase (*KAO*) [4], and their activity is critical for GA-dependent flowering and floral organ development [5]–[9]. Similarly, GA signal transduction factors play important roles in flower development. The GA receptors are encoded by three homologous *GIBBERELLIN-INSENSITIVE DWARF1* (*GID1*) genes (*AtGID1a, AtGID1b* and *AtGID1c*) in *Arabidopsis* [10]. Despite single mutant or double mutants of *gid1* display no or partial GA-deficient phenotypes respectively, triple mutant showed severe GA-deficient abnormality, including extremely dwarfism, delayed flowering, incomplete floral organs and GA-insensitivity [11]. Similarly, in rice, mutation of the GA receptors leads to GA-insensitive and dwarf phenotypes, while overexpression of *GID1* results in early flowering [12]. Another key player in GA signaling pathway is DELLA repressors [13], [14]. Binding of GA to *GID1* promotes the interaction between GID1 and DELLA proteins, which leads to rapid degradation of DELLA proteins through the *SCF^SLY1/GID2^* (*Skp1*, *Cullin*, *F-box* complex) ubiquitin-proteasome pathway, and the proteolysis of DELLA proteins releases their inhibitory effect on GA-responsive genes and allows plant growth and development [1], [15]–[19]. DELLA proteins belong to a subfamily of the GRAS family and have five members in *Arabidopsis*: RGA (REPRESSOR OF *ga1-3*), GAI (GIBBERELLIN INSENSITIVE), RGL1 (RGA-LIKE 1), RGL2 (RGA-LIKE 2), and RGL3 (RGA-LIKE 3) [1], [20]. *RGA* and *GAI* are negative regulators for stem elongation [21]–[23]. *RGA* and *RGL2* coordinately inhibit the development of petal, stamen and anther, while *RGL1* exacerbates this repression [24]–[26]. Transient induction of RGA greatly downregulates the transcription of floral homeotic genes *APETALA3* (*AP3*), *PISTILLATA* (*PI*), and *AGAMOUS* (*AG*), while removing the RGL2 and RGA DELLA activities in *ga1-3* mutant (*ga1-3 rgl2-1 rga-t2*) can rescue the phenotypes of flower development, including delayed flowering time, aberrant petal, stamen and anther development, suggesting that GA regulates flower development via degradation of DELLA proteins, especially RGA and RGL2, thus allows the transcription of floral homeotic genes [21], [24], [27]. *GAMYB*, on the other hand, acts as a positive regulator for GA signaling pathway [28]–[30]. Mutation of the *GAMYB* in *Arabidopsis* (*myb33myb65*) results in shorter filaments, pollen abortion and male sterile, similar to the GA-insensitive phenotype [31]. In rice, *GAMYB* is involved in programmed cell death (PCD) of tapetal cells, exine and ubisch body formation, as well as in the GA-induced anther development [32].

However, so far, most GA-regulated flower development studies were performed in hermaphroditic species, and rarely in monoecious plants. Cucumber (*Cucumis sativus* L.) is a typical monoecious vegetable with individual male and female flowers, and has been served as a model system for sex determination in *planta* [33]. GA can promote male flower formation in cucumber, and the molecular mechanism remains unknown. In this study, we found that cucumber homologs of GA signal transduction factors *GID1*, *DELLA* and *GAMYB* have much higher expression than those of GA synthesis genes during male flower development, and the cucumber *DELLA* homolog *CsGAIP* has the highest expression. We cloned the *CsGAIP* and characterized its spatial and temporal expression patterns. *CsGAIP* is mainly expressed in stems and male flower buds, and CsGAIP protein is located in nucleus. Ectopic expression of *CsGAIP* can partially rescue the phenotypes of *rga-24/gai-t6* double mutant in *Arabidopsis*, and overexpression of *CsGAIP* in wild type resulted in reduced number of stamens and decreased transcription of B class floral homeotic genes. Our results suggested that *CsGAIP* inhibits stamen development through transcriptional repression of B class floral homeotic genes in *Arabidopsis*.

## Results

### Cucumber *DELLA* homolog *GAIP* may have prominent role during male flower development

GA has been shown to promote male flower development in cucumber [3], but the underlining mechanism remains elusive. As the first step to uncover this mystery, we explored the expression patterns of cucumber homologs of GA biosynthesis genes *CPS, KS, KO, KAO* and GA signal transduction factors *GID1*, *DELLA* and *GAMYB* during different stages of male flower development. Using the sequence information in *Arabidopsis*, we performed BLAST analysis in Cucumber Genome Database [34], and defined the best hit as the corresponding cucumber homolog and the relative unique region of each gene was designed for quantitative real-time RT-PCR (qRT-PCR) analyses.

**Figure 1:**
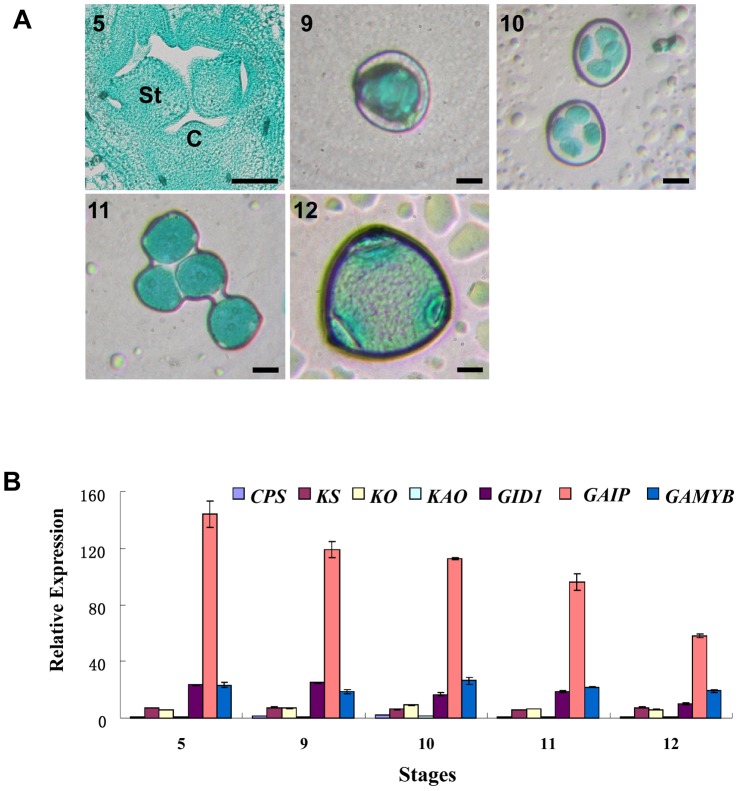
Expression analyses of GA biosynthesis genes and GA signal transduction factors during different developmental stages of cucumber male flowers. (**A**) Light microscopy images of microspores at different developmental stages of cucumber male flowers. Stage 5, hermaphrodite stage; 9, microspore mother cell stage; 10, microspore tetrad stage; 11, early stage of pollen grain development; 12, mature male flower stage. Microspores were stained with carmine and fast green counterstain. St, stamen primordium; C, carpel primordium. Bars = 200 µm. (**B**) qRT-PCR analyses of GA biosynthesis genes and GA signal transduction factors during male flower development. The number 5, 9, 10, 11 and 12 represent the developmental stages. The cucumber *α-TUBULIN* (*TUA*) was used as an internal control, and the experiments were repeated in three independent samples. Error bars indicate the standard errors. *CPS*, copalyl diphosphate synthase; *KS*, ent-kaurene synthase; *KO*, ent-kaurene oxidase; *KAO*, ent-kaurenoic acid oxidase; *GID1*, *GIBBERELLIN-INSENSITIVE DWARF*.

The developmental process of cucumber male flower can be divided into 12 stages [35], in which, five stages including hermaphrodite stage (stage 5), microsporocyte stage (stage 9), meiosis stage (stage 10), uninuclear pollen stage (stage 11) and mature pollen stage (stage 12) were identified based on morphological indications [35], [36] (Figure 1A) and the lengths of cucumber male floral buds for each stage was calculated (Table 1). Then, RNA samples were collected from at least three independent male flower buds and qRT-PCR was performed using these samples. As shown in Figure 1B, GA signal transduction factors *GID1*, *GAIP* (the best hit for *DELLA* homolog) and *GAMYB* have much higher expression than those of GA synthesis genes *CPS*, *KS*, *KO*, *KAO* during cucumber male flower development. In which, *GAIP* has the highest expression among all, particularly in the hermaphrodite stage (stage 5), for example, expression of *GAIP* is more than 20 fold and 6 fold higher than GA synthesis genes and other GA signal transduction factors, respectively. Further, expression of *GAIP* decreases as the male flower develop, suggesting that cucumber *GAIP* may play a key role during male flower development and promote male determination in the hermaphrodite stage.

**Table 1:** Lengths of cucumber male floral buds at different developmental stages.

Developmental stage	Lengths of male floral buds (mm)
Stage 5	1.5±0.3
Stage 9	2.2±0.2
Stage 10	2.9±0.4
Stage 11	4.2±0.6
Stage 12	8.1±0.9

The data in the table can only be used as a reference because the floral length for each developmental stage is affected by variety and environmental conditions. The values shown are the means ± SE of 10 male flowers in the respective stage.

### Cloning and phylogenetic analysis of cucumber *DELLA* homolog *CsGAIP*

**Figure 2:**
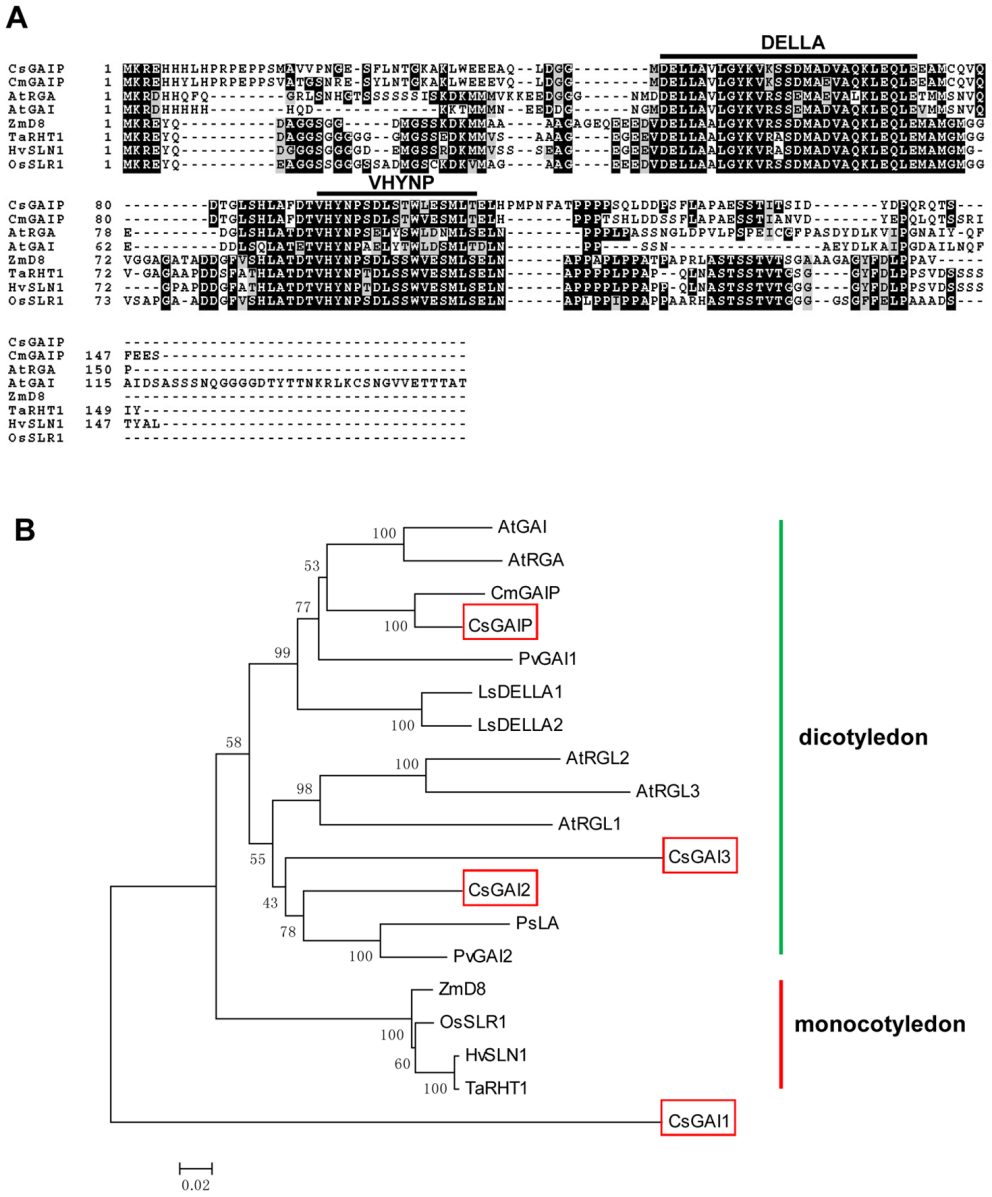
Sequence alignment and phylogenetic analyses of CsGAIP and related DELLA proteins. (**A**) Sequence alignment of the 150 amino acid residues of CsGAIP N-terminal with other DELLA proteins. The identical and similar residues are shown in black and gray, respectively. The highly conserved DELLA and VHYNP domains are indicated in black lines. At, *Arabidopsis thaliana*; Cm, *Cucurbita maxima*; Cs, *Cucumis sativus*; Zm, *Zea mays*; Os, *Oryza sativa*; Hv, *Hordeum vulgare*; Ta, *Triticum aestivum*. (**B**) Phylogenetic analyses of CsGAIP and related DELLA proteins using MEGA5 software based on the neighbor joining method. Homologs of DELLA from six dicotyledon species (green line) and four monocotyledon species (red line) were used for the analyses and formed distinct clade (dicotyledon group and monocotyledon group). The four DELLA homologs from cucumber are indicated in red boxes. Gene ID for each of the DELLA protein used for this analysis is listed in the “accession numbers”. Ls, *Lactuca sativa*; Ps, *Pisum sativum*; Pv, *Phaseolus vulgaris*.

Through BLAST analysis, we found four *DELLA* homologs in cucumber, *CsGAIP* (*Csa021618*), *CsGAI1* (*Csa015919*), *CsGAI2* (*Csa008181*) and *CsGAI3* (*Csa015258*), in which *CsGAIP* has the highest similarity to *DELLAs* in *Arabidopsis*, so we chose *CsGAIP* for further analysis in this study. *CsGAIP* was cloned using cDNA derived from cucumber leaves through PCR technology. The full-length *CsGAIP* cDNA consists of 1761 bp and encodes 587 amino acids. Consistent with the five *DELLA* genes of *Arabidopsis*, *CsGAIP* gene also has no introns [13], [14]. Previous studies showed that DELLA proteins belong to a GRAS subfamily that contains two highly conserved domains named as DELLA and VHYNP in their N-terminal regions [14], [22], [37]. Sequence alignment of the N-terminal 150 amino acid residues of CsGAIP using ClustalW indicated that CsGAIP also has the DELLA and VHYNP domains, which may be essential for GID1-DELLA interaction [11], [38]–[42] (Figure 2A). Full-length CsGAIP is 89.25%, 64.72%, 64.91%, 53.28%, 51.96%, 52.53%, 52.9% identical to CmGAIP, AtRGA, AtGAI, ZmD8, TaRHT1, HvSLN1, OsSLR1, respectively. To understand the evolutional relationship between CsGAIP and other DELLA proteins, we constructed phylogenetic tree using the neighbor-joining (NJ) method [43] (Figure 2B), cucumber CsGAIP, CsGAI2 and CsGAI3 are placed in the same cluster as other DELLA homologs, while CsGAI1 is distantly related, suggesting that CsGAIP, CsGAI2 and CsGAI3 are more likely to be the DELLA homologs in cucumber. Phylogenetic tree divides DELLA homologs into two clades: dicotyledon (green line) such as *Arabidopsis*, cucumber, pumpkin (*Cucurbita maxima*), lettuce (*Lactuca sativa*), pea (*Pisum sativum*), bean (*Phaseolus vulgaris*), and monocotyledon (red line) such as maize, rice, barley and wheat. Within dicotyledon clade, CsGAIP and CmGAIP, which belong to the cucurbitaceae family with unisexual flowers, fall into the same clade that is distinct from those of CsGAI2, CsGAI3 and other DELLA homologs in hermaphroditic species, such as *Arabidopsis*, lettuce, pea and bean, implying that CsGAIP may be involved in the unisexual flower development in cucumber.

### Expression pattern of *DELLA* homologs in cucumber

**Figure 3:**
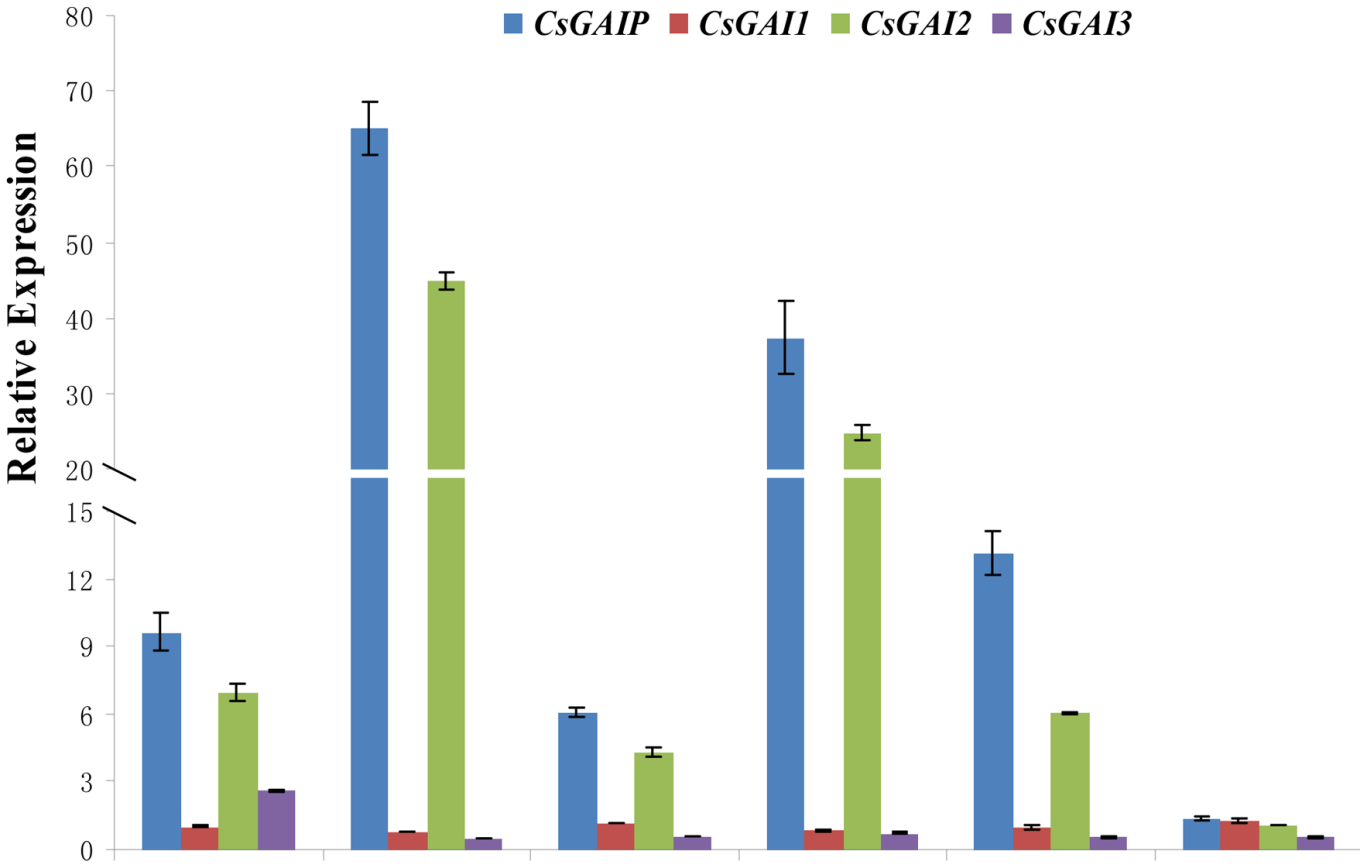
qRT-PCR analyses of four *DELLA* homologs in different tissues of cucumber. Three biological replicates were performed for this experiment and the cucumber *TUA* gene was used as an internal control. Error bars indicate the standard errors. R, roots; S, stems; L, leaves; MB, male flower buds; FB, female flower buds; F, fruits.

To characterize the spatial distribution of *DELLA* homologs transcripts, qRT-PCR was performed in various cucumber tissues including roots, stems, leaves, male flower buds, female flower buds and fruits. As shown in Figure 3, expressions of *CsGAIP* and *CsGAI2* are much higher than those of *CsGAI1* and *CsGAI3* in all the tissues we examined, and that *CsGAIP* and *CsGAI2* display similar expression patterns, which are predominantly expressed in stems and male flower buds. *CsGAI3* transcript is more enriched in roots as compared to other tissues, while *CsGAI1* shows equivalent expression in all the tissues we tested. Among all the four *DELLA* homologs, *CsGAIP* displays the highest expression especially in stems and male flower buds, implicating that *CsGAIP* may play important roles in stem and male flower development.

**Figure 4:**
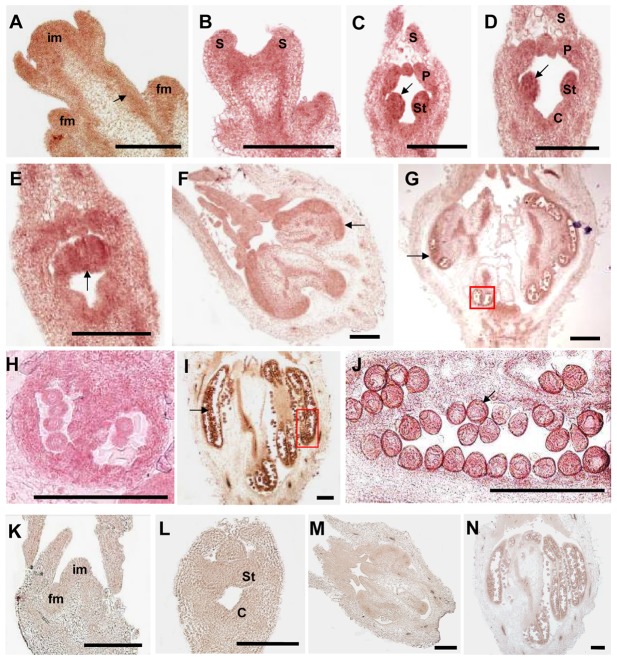
*In situ* hybridization of *CsGAIP* during male flower development in cucumber. Longitudinal sections of the shoot apex (A and K, early stage 1) and male flower buds at stage 2 (B), stage 4 (C), stage 5 (D), stage 6 (E and L), stage 9 (F and M), stage 11 (G) and stage 12 (I and N). The pollen morphology in the framed regions of G and I was shown in H and J, respectively. *CsGAIP* sense probe hybridizations showed no signals in K–N. The arrow in A indicated the vascular expression of *CsGAIP*, and the arrows in C–J showed the strong expression of *CsGAIP* in developing stamen or pollens. im, inflorescence meristem; fm, floral meristem; S, sepal; P, petal; St, stamen; C, carpel. Bar = 200 µm.

Next, we examined the expression pattern of *CsGAIP* during male flower development of cucumber by *in situ* hybridization (Figure 4). *CsGAIP* RNA was found throughout in the inflorescence meristem (im) and floral meristem (fm) (Figure 4A), as well as in the vascular strands (arrow in Figure 4A) in stage 1 male flowers [35]. During stages 2–6 (hermaphrodite stage), *CsGAIP* is expressed in the developing sepals, petals, stamens and carpels, with the strongest expression in stamen primordia (arrows in Figure 4B–E). As the male flower further develop, microsporocytes initiate in stage 9, uninuclear pollen appear in stage 11 and mature pollen form by stage 12, and *CsGAIP* is expressed mainly in the microsporocytes (Figure 4F), anther wall and pollen grains (Figure 4G–J), despite the signal is weaker than those in hermaphrodite stage. This data is consistent with the higher expression in hermaphrodite stage as detected by qRT-PCR (Figure 1B). As negative controls, *CsGAIP* sense probe hybridizations show no signals in male flowers of stage 1, stage 6, stage 9 and stage 12 (Figure 4K–N).

### Subcellular localization of CsGAIP

**Figure 5:**
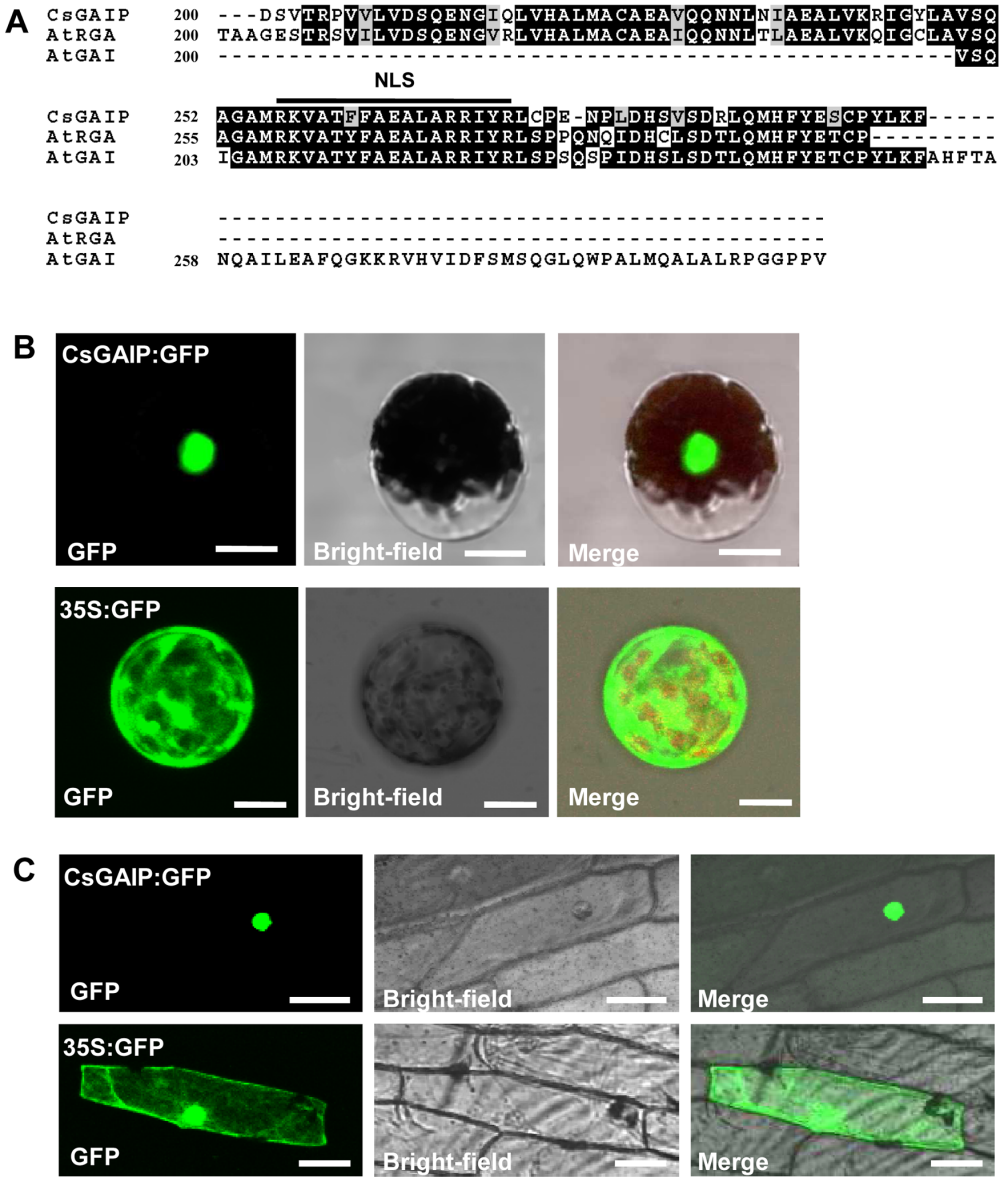
Subcellular localization of CsGAIP protein. (A) Alignment of the N-terminal 200–300 amino acid residues of CsGAIP with AtRGA and AtGAI. The black line indicates the highly conserved nuclear localization signal (NLS) domain. (B) Subcellular localization of CsGAIP protein in cucumber protoplasts. *35S:GFP-CsGAIP* (full length *CsGAIP* fused with GFP protein) localized to the nucleus, while *35S:GFP* (GFP protein driven by 35S promoter) localized throughout the cell. Bar = 20 µm. (C) Subcellular localization of CsGAIP protein in onion epidermal cells. *35S:GFP-CsGAIP* localized to the nucleus, and *35S:GFP* control localized throughout the cell. Bar = 50 µm.

In *Arabidopsis*, the DELLA proteins RGA and GAI have been shown to contain putative nuclear localization signal (NLS) and localize in nucleus [14]. Sequence alignment of the N-terminal 200–300 amino acid residues of CsGAIP with AtRGA and AtGAI showed that CsGAIP also has a putative NLS (Figure 5A). Subcellular localization of CsGAIP in cucumber protoplasts indicated that CsGAIP locates in nucleus as well (Figure 5B, top row), and the same result was found in epidermal cells of onion (*Allium cepa*) (Figure 5C, top row). As a control, signals of 35S:GFP were detected throughout the cell (Figure 5B and C, bottom row).

### *CsGAIP* can partially rescue *rga-24/gai-t6* double mutant in *Arabidopsis*

**Figure 6:**
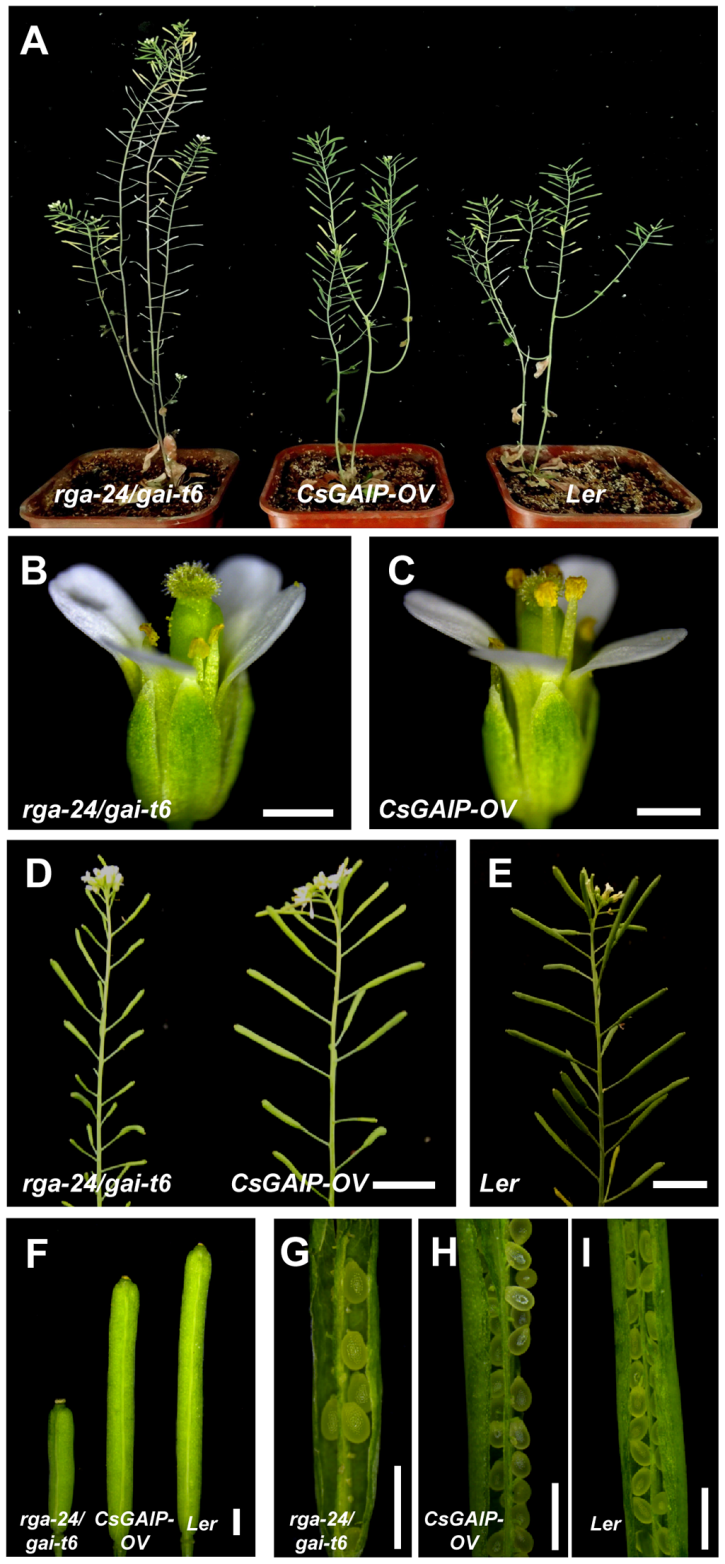
Partial rescue of *rga-24/gai-t6* mutant by ectopic expression of *CsGAIP* in *Arabidopsis*. (A) Plant heights of *rga-24/gai-t6* (left), *CsGAIP* overexpression (middle) or *Ler* (right) of 58 days old. (B–C) Flowers of *rga-24/gai-t6* (B) or *CsGAIP* overexpression (C). (D–E) Inflorescences of *rga-24/gai-t6* (D, left), *CsGAIP* overexpression (D, right) or *Ler* (E). (F) Siliques of *rga-24/gai-t6* (left), *CsGAIP* overexpression (middle) or *Ler* (right). (G–I) Opened siliques of *rga-24/gai-t6* (G), *CsGAIP* overexpression (H) or *Ler* (I) at similar developmental stage. Bar = 1 mm, except D and E, in which Bar = 1 cm.

To explore the function of *CsGAIP*, we ectopically expressed the full-length *CsGAIP* cDNA under the control of 35S promoter of *Cauliflower mosaic virus* (CaMV) in *Arabidopsis rga-24/gai-t6* double mutant, and 13 independent transgenic lines were obtained. Previous study reported that *rga-24/gai-t6* double mutant displayed higher plant height, reduced number of pollens, shorter filaments and thus decreased seed numbers per silique [21]. As showed in Figure 6 and Table 2, all the transgenic lines display partial rescue of the *rga-24/gai-t6* phenotypes. The average plant height of *rga-24/gai-t6* plants is 38% taller than that of *Ler*, while in the transgenic lines, the average plant height is only 8% taller than that of *Ler* (Fig. 6A; Table 2), suggesting that *CsGAIP* can greatly rescue the plant height phenotype in *Arabidopsis*. Further, flowers in the *CsGAIP* transgenic plants display increased filaments length and amount of pollen as compared to those in *rga-24/gai-t6* (Fig. 6B, C). Consequently, the silique length and the seed number per silique increase in the transgenic plants (Figure 6D–I). For example, there are around 8 seeds per silique in the *rga-24/gai-t6* plant, while ectopic expression of *CsGAIP* in *rga-24/gai-t6* background results 43 seeds/silique, which is close to that in *Ler* (56 seeds/silique) (Table 2). These data suggested that cucumber *CsGAIP* can partially replace the function of *RGA* and *GAI* in *Arabidopsis* with respect to plant height, stamen development and plant fertility.

**Table 2:** *CsGAIP* can rescue the plant height and fertility of *rga-24/gai-t6* in *Arabidopsis*.

Genotype	Number of plants	Plant height (cm)	Seeds/silique^1^
*rga-24/gai-t6*	13	26.1±0.8 a	8.0±1.6 a
*35S:CsGAIP rga-24/gai-t6*	13	20.5±0.8 b	43.4±4.0 b
*Ler*	13	18.9±1.1 c	56.4±3.2 c

The values shown are the means ± SE of 13 plants from *rga-24/gai-t6*, 13 *CsGAIP* transgenic T1 lines or 13 *Ler* plants, respectively. Different letters (a–c) in the same column indicate significant differences (*P*<0.05) determined by Duncan's test.

1 Fertility was counted by the number of seeds per silique. Ten siliques were measured in each plant.

### *CsGAIP* suppresses stamen development by down-regulating floral homeotic genes *AP3* and *PI* in *Arabidopsis*

**Figure 7:**
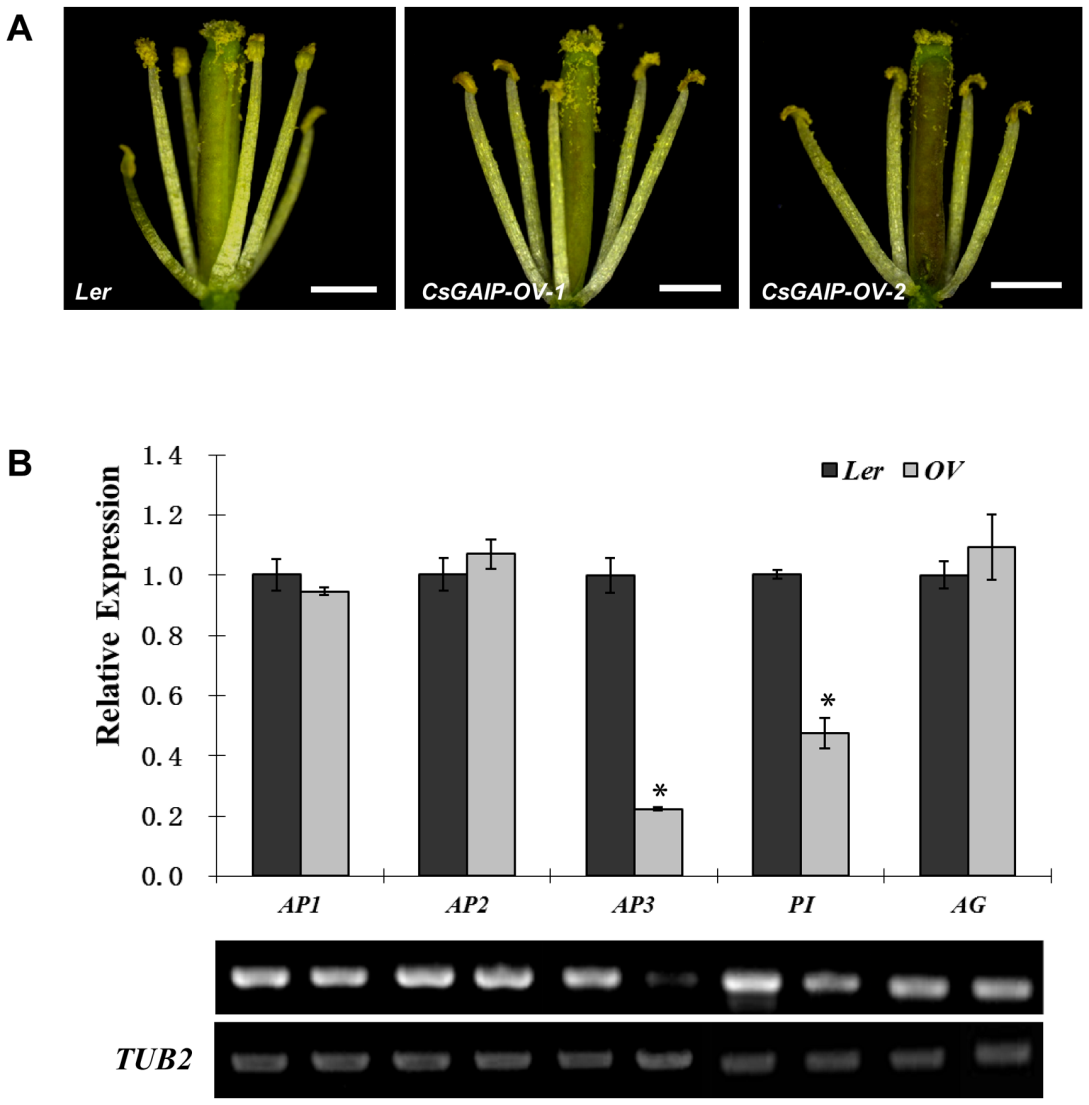
Transcription analyses of floral homeotic genes upon ectopic expression of *CsGAIP* in WT *Arabidopsis*. (A) Stamens in *Ler* or lines of *CsGAIP* overexpression. (B) qRT-PCR (top) and semi-quantitative RT-PCR (bottom) analyses of floral homeotic genes in the inflorescence apices of *Ler* or *CsGAIP* overexpression lines. The β-*tubulin* gene (*TUB2*) was used as an internal control, and three biological replicates were performed for each gene. Asterisks indicate the significant differences (*P*<0.01) between *Ler* and *CsGAIP* overexpression lines determined by Duncan's test.

We further explore the function of *CsGAIP* by overexpression of *CsGAIP* in *Arabidopsis* wide-type *Ler*, and 25 independent transgenic lines were obtained. As shown in Figure 7A, ectopic expression of *CsGAIP* in *Arabidopsis* led to reduced number of stamens. In contrast to the six stamens in *Ler* flowers, the flowers in *35S::CsGAIP* plants only display 4.6±0.5 stamens (Table 3). Given that the floral homeotic genes, including *APETALA1 (AP1)*, *APETALA2 (AP2)*, *APETALA3 (AP3)*, *PISTILLATA* (*PI*) and *AGAMOUS (AG)*, are involved in floral patterning in *Arabidopsis* [44], and that B genes (*AP3* and *PI*) and C gene (*AG*) are down-regulated by RGA activity [27], we examined the expression of floral homeotic genes in *35S::CsGAIP* plants by qRT-PCR and semi-quantitative RT-PCR. We found that the expression of A class (*AP1* and *AP2*) and C class of gene (*AG*) were not substantially changed in the transgenic plants, but transcripts of B function genes (*AP3* and *PI*) were significantly decreased (Fig. 7B). For example, the transcripts of *AP3* and *PI* in the *35S*::*CsGAIP* plants were reduced by around 80% and 50% respectively as compared to those in the *Ler* background. These data suggested that *CsGAIP* can suppress the expression of B function genes in *Arabidopsis*, which may be the cause for reduced number of stamens as observed in the ectopic expression lines.

**Table 3:** Reduced numbers of stamens upon overexpression of *CsGAIP* in *Arabidopsis*.

Genotype	Number of plants	Number of stamens^1^
*Ler*	10	6.0±0.0 a
*35S:CsGAIP Ler*	25	4.6±0.5 b

The values shown are the means ± SE of 25 *CsGAIP* transgenic T1 plants, or 10 *Ler* plants. Different letters (a and b) in the same column indicate significant differences (*P*<0.05) between *Ler* and transgenic plants determined by Duncan's test.

1 The number of stamens were the average of 20 flowers from each line.

## Discussion

Cucumber (*Cucumis sativus* L.) is a monoecious species with individual male and female flowers. During the early stage of flower development, both stamen primordia and carpel primordia are initiated, male or female flower is generated upon the arrestment of carpel or stamen development, respectively [33], [35]. Due to the agricultural importance, extensive studies have been performed in the mechanism of female flower formation, while the molecular regulation of male flower generation is largely unknown [45]–[51]. GA can regulate flower development in both hermaphroditic and monoecious species [3]. In *Arabidopsis*, GA promotes stamen development by antagonizing the function of DELLA proteins [24]. In monoecious cucumber, how GA stimulates male flower development remain elusive. Here we found that the cucumber *DELLA* homolog may play important roles during male flower development in cucumber (Figure 1, Table 1), and we cloned this *DELLA* homolog *CsGAIP* (Figure 2) and investigated the expression pattern and subcellular localization (Figure 3–5). Further, we explored the function of *CsGAIP* through ectopic overexpression of *CsGAIP* in *Arabidopsis* (Figure 6 and 7, Table 2 and 3). Our data suggested that monoecious *CsGAIP* may repress staminate development through transcriptional downregulation of B class floral homeotic genes *in Arabidopsis*.

### *CsGAIP* may be the homolog for both *RGA* and *GAI* in cucumber

In *Arabidopsis*, *DELLA* family has five members: *RGA*, *GAI*, *RGL1*, *RGL2*, and *RGL3* [20], which coordinately function in stem elongation, floral organ development and flowering [21], [23]–[26]. In cucumber, there are four putative *DELLA* genes, *CsGAIP* (*Csa021618*), *CsGAI1* (*Csa015919*), *CsGAI2* (*Csa008181*) and *CsGAI3* (*Csa015258*), with *CsGAIP*, *CsGAI2* and *CsGAI3* closely relate to *RGA* and *GAI*, and *CsGAI1* likely to be the homolog for *RGL1-3* (Figure 2, data not shown). Phylogenetic analyses indicate that CsGAIP but not CsGAI2 or CsGAI3 falls into the same clade as RGA and GAI (Figure 2). Further, similar to those of RGA and GAI, CsGAIP has a NLS domain and localizes to nucleus (Figure 5), suggesting that *CsGAIP* may be the homolog for both *RGA* and *GAI*. *RGA* and *GAI* have been shown to be negative regulators for stem elongation and stamen development, in which *RGA* inhibits stamen development via repressing floral homeotic genes *AP3*, *PI*, and *AG* [21], [24], [27]. Similarly, *CsGAIP* is highly expressed in stem and male flower buds, and ectopic expression of *CsGAIP* can partially rescue the plant height, stamen development and fertility phenotypes of *rga-24/gai-t6* double mutant (Figure 6), and that overexpression of *CsGAIP* in wide-type *Arabidopsis* leads to decreased transcription of *AP3* and *PI* (Figure 7). These data suggested that *CsGAIP* may functions as the homolog of both *RGA* and *GAI* in cucumber.

### *CsGAIP* may inhibit male tendency during sex determination of cucumber flowers

In cucumber, exogenous GA treatment can promote male flower formation [52], [53]. In this study, we found that *CsGAIP* is predominantly expressed in the male specific organs during flower development of cucumber, particularly in stamen primordia (Figure 1, 3 and 4). Ectopic expression of *CsGAIP* results in transcriptional repression of B class floral homeotic genes *AP3* and *PI* in *Arabidopsis*. Therefore, we propose that *CsGAIP* may function as a major repressor for GA-induced male flower tendency. During the hermaphrodite stage, there may be equal activities for male-promoting and female-promoting factors, male and female flowers are produced by random with similar chance. Exogenous GA application may promote the interaction between GA receptors and CsGAIP, which may lead to rapid proteolysis of CsGAIP protein through the *SCF^SLY1/GID2^* ubiquitin-proteasome pathway. Such CsGAIP degradation can stimulate the transcription of B class floral homeotic genes and thus promote staminate (male flower) development. Genetic transformation in cucumber upon *CsGAIP* RNA interference or overexpression would shed light on the molecular function of *CsGAIP* during sex determination of cucumber flowers. Meanwhile, *F* (*CsACS1G*) and *M* (*CsACS2*) genes have been demonstrated to regulate unisexual flower development in cucumber, specifically, *F* gene promotes female flower development [50], [54], [55], and *M* gene inhibits stamen development in floral buds [47]–[49]. It would be interesting to dissect the interactions, if any, between *CsGAIP, F* and *M* during sex determination in future studies. In addition, in monoecious maize, GA causes feminization instead of staminate production [56], implying that distinct mechanisms may be involved in the GA-mediated flower development in different species.

### Unisexual *CsGAIP* displays conserved as well as divergent functions with its bisexual homologs

Loss of function of *RGA* and *GAI* in *Arabidopsis* results in higher plant height and earlier flowering [21], while lack of *DELLA* homologs *REDUCED HEIGHT* and *DWARF8* leads to dwarfism in wheat and maize, respectively [37], [57]–[59], indicating that *DELLA* homologs have conserved role in stem elongation, but the specific role maybe even opposite in different species. In this study, *CsGAIP* is highly expressed in cucumber stems (Figure 3) and that *CsGAIP* can rescue the plant height phenotype of *rga-24/gai-t6* (Fig. 6, Table 2), suggesting that *CsGAIP* may also function as a suppressor for stem elongation as those of *Arabidopsis RGA* and *GAI*. Similarly, transcripts of *CsGAIP* are enriched in stamen primordia, and ectopic expression of *CsGAIP* can rescue the stamen development and plant fertility phenotypes in *rga-24/gai-t6* (Figure 6, Table 2), and lead to reduced number of stamens and decreased expression of B function genes *AP3* and *PI* upon ectopic expression in *Ler* (Figure 7, Table 3), supporting that *CsGAIP* has a conserved role in flower development, specifically, inhibits staminate development via repressing B class of floral homeotic genes. However, unlike the down-regulating of both B and C function genes upon RGA induction in *Arabidopsis* [27], the transcription of C class gene *AG* remains unchanged upon ectopic expression of *CsGAIP* (Figure 7B), similarly, flowering time appeared to be undisturbed upon overexpression of *CsGAIP* in *Arabidopsis* (data not shown), suggesting that monoecious *CsGAIP* has divergent functions from *RGA* and *GAI* in hermaphroditic *Arabidopsis*.

Given that *Arabidopsis DELLAs* have specific as well as partially overlapping roles, it would be interesting to explore the specificity of the function for each DELLA homologue in cucumber. The four cucumber *DELLAs* display distinct expression patterns (Figure 3), in which *CsGAI1* has low transcript accumulation in all the tissues we examined, *CsGAI3* is predominantly expressed in roots, whereas *CsGAIP* and *CsGAI2* are highly enriched in stems and male flower buds, suggesting that *CsGAIP* and *CsGAI2* may play important and probably partially redundant roles in stem and male flower development in cucumber. However, for elucidating the functional similarities and differences among these four *DELLAs*, cucumber transformation, a currently difficult technique, is the best way to uncover the mystery in future studies. In addition, given that DELLA can regulate the cross-talks between GA and other signaling pathways through protein-protein interactions with regulatory factors such as PIF3/PIF4 (PHYTOCHROME-INTERACTING FACTOR 3/4), SCL3 (SCARECROW-LIKE 3), ALC (ALCATRAZ) and JAZs (JA ZIM-domain proteins) [18], [60], identifying the DELLA interacting proteins will greatly advance our knowledge of the diverse functions of DELLA homologs in cucumber development.

## Materials and Methods

### Plant materials and growth conditions

A monoecious cucumber (*Cucumis sativus L.*) line 3461 was used in this study. The plants were grown in a growth chamber under 16 h/8 h and 25°C/18°C in day/night, respectively. Upon two true-leaf stage, plants were transferred to a greenhouse in the experimental field of China Agricultural University in Beijing. The *Arabidopsis* mutant *rga-24/gai-t6* (*Landsberg* background) was provided by Sun's lab [21], and *Ler* was used as wild type control. *Arabidopsis* seeds were germinated on Murashige-Skoog (MS) medium, which contains 1% sucrose and 0.2% phytagar at 4°C for 3 days and then moved to 22°C under a regime of 16 h light/8 h dark. Seedlings were transferred to soil 7–10 d after germination.

### Cloning of *CsGAIP*, sequence alignment and phylogenetic analysis

Total RNA was extracted from cucumber leaves using the Promega's SV Total RNA Isolation System, and cDNA was synthesized using MultiScribe reverse transcriptase (Applied Biosystems). The cDNA was amplified with primers CsGAIP-F (5′-ATGAAGAGGGAGCATCACCATCTTC-3′) and CsGAIP-R (5′-TCACTTAGCGACCACCGGGTT-3′) at 95°C for 5 min; 30 cycles of 95°C for 30 s, 54°C for 30 s, and 72°C for 2.5 min; and then 72°C for 10 min. The amino acid sequence of related DELLA proteins were obtained from National Center for Biotechnology Information (http://www.ncbi.nlm.nih.gov) or the Arabidopsis Information Resource (http://www.arabidopsis.org), and protein alignment of *CsGAIP* and related DELLAs was performed using ClustalW in the MEGA5 software package, and the boxes were drawn using the BoxShade web site (http://www.ch.embnet.org/software/BOX_form.html). The phylogenetic tree was constructed using the neighbor-joining (NJ) method [43] through MEGA5 software using the bootstrap analysis with1000 replications.

### Gene expression analysis

Total RNA was extracted using Promega's SV Total RNA Isolation System, and cDNA was synthesized using MultiScribe reverse transcriptase (Applied Biosystems). Quantitative real-time RT-PCR (qRT-PCR) was performed using SYBR *Premix Ex Taq* from TaKaRa (China) on an Applied Biosystems 7500 real-time PCR system. The cucumber *α-tubulin* gene (*TUA*) and *Arabidopsis β-tubulin* gene (*TUB2*) were used as internal references. For semi-quantitative RT-PCR, the *β-tubulin* gene (*TUB2*) was used as a control. Both qRT-PCR and semi-quantitative PCR were repeated in three independent samples. The gene primers for qRT-PCR were as follows: CPS-F (5′-GCTGAGGTCAATGGACGATG-3′) and CPS-R (5′-TGAGAATATTTGACTGTCACCCC-3′); KS-F (5′-CAATGGTCCCTTCTCCAAACT-3′) and KS-R (5′-CCCATCGCTTAAGAGTAAGAACAC-3′); KO-F (5′-AAGAGGCTAT- GGTGACGAGGTA-3′) and KO-R (5′-ACATGAGCAAACAACTCCCTAGA-3′); KAO-F (5′-CACTCAAGGCTCGGAAGAATC-3′) and KAO-R (5′-CAACATCAATCAGAGCGTCCAT-3′); GID1-F (5′-ATCCAGCATGTAATCCCTTCG-3′) and GID1-R (5′-CCATCATTCTCCAGCCCTCT-3′); CsGAIP-F (5′-GCTCAAACGCATTCAAACAAG-3′) and CsGAIP-R (5′-GCTATGAGTGGGCGAGTGTG-3′); CsGAI1-F (5′-GCCGTCCACTACAACCCTTCC-3′) and CsGAI1-R (5′-GTCCACGAGACACTCCCATCC-3′); CsGAI2-F (5′-TAAAGACGACGAAGCCGAAGATA-3′) and CsGAI2-R (5′-AATAAACCTCCGACAACAACACG-3′); CsGAI3-F (5′-GGAGGAAGACCACGACAAGCATC-3′) and CsGAI3-R (5′-CGGAGTATTGAGTTCAGCGAGCA-3′); GAMYB-F (5′-TCTAACCCTACCACAAAGAACGC-3′) and GAMYB-R (5′-TCTATCTGGTGCCAACACAAAAGT-3′); TUA-F (5′-ACGCTGTTGGTGGTGGTAC-3′) and TUA-R (5′-GAGAGGGGTAAACAGTGAATC-3′); AP1-F (5′-GTTGCTCTTGTTGTCTTCTCCC-3′) and AP1-R (5′-CTCCATCGACCAGTTTGTATTG-3′); AP2-F (5′-GGTGTTGCTTCTGGCTTTCCT-3′) and AP2-R (5′-GTCCACGCCGACTCTTTTTCA-3′); AP3-F (5′-TATTTCTGATGTCGATGTTTGGGC-3′) and AP3-R (5′-ACTTTTGTTCTTTTTCTTGGTGGT-3′); PI-F (5′-TGGATTGGTGAAGAAGGCTAA-3′) and PI-R (5′-GATCTCCATCTGGTGGTCTCG-3′); AG-F (5′-ATAATCAGCATACAAAACTCCAAC-3′) and AG-R (5′-ATACTTCTCTCTAATCTGCCTTCC-3′); TUB2-F (5′-ATCCGTGAAGAGTACCCAGAT-3′) and TUB2-R (5′-AAGAACCATGCACTCATCAGC-3′). The primers of *AP1*, *AP2*, *AP3*, *PI* and *AG* for semi-quantitative PCR were performed as previously reported [27].

### *In situ* hybridization

Shoot apex of 10-day-old seedling and male flower buds from 45-day-old cucumbers grown in the greenhouse were fixed and hybridized as described [61]. Digoxigenin-labeled probes were generated through PCR amplification of cDNA using gene specific primers containing SP6 and T7 RNA polymerase-binding sites. SP6 and T7 RNA polymerase were used for the synthesis of sense and antisense probes, respectively. The primers of cucumber *CsGAIP in situ* probes were as follow: 5′-**GATTTAGGTGACACTATAGAATGCT**ATCCGATGCCTAATTTTGCGA-3′ (bold represents the SP6 RNA polymerase binding sites) and 5′-**TGTAATACGACTCACTATAGGG**GCATCTGAAGCCTATCGGACACT-3′ (bold shows the T7 RNA polymerase binding sites).

### Subcellular localization in cucumber protoplasts and onion epidermal cells

For transient expression in cucumber protoplasts and **onion epidermal cells**, the full length coding region of *CsGAIP* were cloned using primers 5′-ACGC**GTCGAC**ATGAAGAGGGAGCATCACCATCTTC-3′ (Sal I site in bold) and 5′-CG**GGATCC**CTTAGCGACCACCGGGTTGTT-3′ (BamH I site in bold), and then inserted into the pEZS-NL vector (with GFP protein driven by 35S promoter) to generate *35S:GFP-CsGAIP*, and the empty pEZS-NL vector was used a control. The constructs were introduced into cucumber protoplasts using Huang's method [62]. The onion epidermal layers were prepared and bombarded, as previously described [63], with gold particles containing the plasmid using a Bio-Rad PDS-1000/He particle delivery system. After bombardment, the onion epidermises were placed on MS medium and incubated in darkness at 22°C for 24 h. Fluorescence signals were detected using Olympus BX 51 fluorescence microscopy.

### Ectopic expression of *CsGAIP* in *Arabidopsis*

To make the *CsGAIP* overexpression construct, full length *CsGAIP* cDNA were cloned using primers 5′-GG**ACTAGT**ATGAAGAGGGAGCATCACCATCTTC-3′ (Spe I site in bold) and 5′-GACTGC**CACG TG**TCACTTAGCGACCACCGGGTT-3′ (Pml I site in bold), and inserted into the pCAMBIA1305.1 vector with 35S promoter. The construct was then introduced into Agrobacterium by electroporation and transformed into Ler or *rga-24/gai-t6* plants as described [64]. The transgenic plants were screened on MS medium with 25 mg/L hygromycin.

### Accession numbers

Sequence data in this study can be found in the Cucumber Genome DataBase, Arabidopsis Genome Initiative or GenBank/EMBL/Swiss-Prot databases under the following accession numbers: *CsGAIP* (Csa021618), *CsGAI1* (Csa015919), *CsGAI2* (Csa008181), *CsGAI3* (Csa015258), *AtRGA* (At2g01570), *AtGAI* (At1g14920), *AtRGL1* (At1g66350), *AtRGL2* (At3g03450), *AtRGL3* (At5g17490), *CmGAIP* (Q6EI06), *ZmD8* (Q9ST48), *TaRHT1* (Q9ST59), *HvSLN1*(Q8W127), *OsSLR1*(Q7G7J6), *LsDELLA1* (BAG71200), *LsDELLA2* (BAG71201), *PsLA* (ABI30654), and *PvGAI2* (BAF62637).
